# Automatic Planning Tools for Lumbar Pedicle Screws: Comparison and Validation of Planning Accuracy for Self-Derived Deep-Learning-Based and Commercial Atlas-Based Approaches

**DOI:** 10.3390/jcm12072646

**Published:** 2023-04-02

**Authors:** Moritz Scherer, Lisa Kausch, Akbar Bajwa, Jan-Oliver Neumann, Basem Ishak, Paul Naser, Philipp Vollmuth, Karl Kiening, Klaus Maier-Hein, Andreas Unterberg

**Affiliations:** 1 Department of Neurosurgery, Heidelberg University Hospital, 69120 Heidelberg, Germany; 2 Division of Medical Image Computing, German Cancer Research Center (DKFZ) Heidelberg, 69120 Heidelberg, Germany; 3 Department of Neuroradiology, Heidelberg University Hospital, 69120 Heidelberg, Germany; 4 Pattern Analysis and Learning Group, Department of Radiation Oncology, Heidelberg University Hospital, 69120 Heidelberg, Germany

**Keywords:** pedicle screw accuracy, deep-learning, machine-learning, atlas planning, spinal navigation, robotic surgery, spinal instrumentation

## Abstract

Background: This ex vivo experimental study sought to compare screw planning accuracy of a self-derived deep-learning-based (DL) and a commercial atlas-based (ATL) tool and to assess robustness towards pathologic spinal anatomy. Methods: From a consecutive registry, 50 cases (256 screws in L1-L5) were randomly selected for experimental planning. Reference screws were manually planned by two independent raters. Additional planning sets were created using the automatic DL and ATL tools. Using Python, automatic planning was compared to the reference in 3D space by calculating minimal absolute distances (MAD) for screw head and tip points (mm) and angular deviation (degree). Results were evaluated for interrater variability of reference screws. Robustness was evaluated in subgroups stratified for alteration of spinal anatomy. Results: Planning was successful in all 256 screws using DL and in 208/256 (81%) using ATL. MAD to the reference for head and tip points and angular deviation was 3.93 ± 2.08 mm, 3.49 ± 1.80 mm and 4.46 ± 2.86° for DL and 7.77 ± 3.65 mm, 7.81 ± 4.75 mm and 6.70 ± 3.53° for ATL, respectively. Corresponding interrater variance for reference screws was 4.89 ± 2.04 mm, 4.36 ± 2.25 mm and 5.27 ± 3.20°, respectively. Planning accuracy was comparable to the manual reference for DL, while ATL produced significantly inferior results (*p* < 0.0001). DL was robust to altered spinal anatomy while planning failure was pronounced for ATL in 28/82 screws (34%) in the subgroup with severely altered spinal anatomy and alignment (*p* < 0.0001). Conclusions: Deep learning appears to be a promising approach to reliable automated screw planning, coping well with anatomic variations of the spine that severely limit the accuracy of ATL systems.

## 1. Introduction

Pedicle screws for posterior instrumentation is a routine procedure in spine surgery. The increased use of navigation for pedicle screw placement has significantly contributed to the safety profile of the procedure by reducing the risk for severe screw misplacement and resulting neuronal or vascular injury in recent years [1,2]. Moreover, the role of screw dimensions and placement accuracy for optimization of construct strength has been pointed out in complex surgical cases with compromised bone quality [3,4,5]. In this regard, dedicated planning of screw dimensions and trajectories prior to the procedure should be pursued to tap the full potential of navigation systems and to achieve optimal results. Similarly, screw planning becomes mandatory in robot-assisted surgeries for communicating desired screw trajectories to the robot [6]. 

Since manual screw planning is a time-consuming procedure on navigation or robotic workstations, several image-based approaches have been described in the literature to facilitate and expedite pedicle screw planning. These comprise detection of geometrical and structural landmarks in spinal imaging or involve registration to anatomical or screw trajectory atlases to derive screw paths and dimensions [7,8,9,10]. 

Machine learning was used in one approach for vertebra segmentation and pedicle identification and provided screw suggestions [10]. However, anatomic variations, previous surgeries and altered spinal alignment were mutual impediments to image-based approaches substantially influencing planning accuracy. Consequently, this limited clinical applicability since those conditions are common findings in everyday spine surgery. 

Our group previously described a novel approach to pedicle screw planning using deep learning. Leveraging a large dataset of virtually planned screws enabled the prediction of screw dimensions and trajectories from the context of unlabeled spinal images using a nnU-net. While proposed screws were noninferior to manual reference screws in the initial validation, robustness to variations in spinal anatomy and alignment was not tested in particular [11]. 

In this study, we aimed to compare the self-derived deep-learning-based (DL) tool for pedicle screw planning to a commercially available atlas-based (ATL) approach and validate results using manually planned reference screws by spine surgeons. Furthermore, our focus was on evaluating the robustness of automatic planning tools to different spinal pathologies, anatomic variations and spinal alignment.

## 2. Materials and Methods

### 2.1. Cases and Study Design

This study was designed as an ex vivo analysis of screw planning tools processing existing data of lumbar and sacral instrumentations from real clinical cases. 

The institutional review board approved the processing of anonymized data for evaluation of screw accuracy in spinal instrumentation and the requirement for informed consent was waived (S-723/2017).

Data was retrieved from a consecutive institutional registry of CT-navigated spinal instrumentations performed at the authors’ institution between January 2010 and December 2018 (*n* = 1660). The registry contained instrumentation surgeries for various indications ranging from fracture stabilization and degenerative spine disease to adult spondylolisthesis and degenerative spinal deformity. Juvenile idiopathic deformity was not evaluated in this study. 

In total, *n* = 50 cases were randomly selected for this study. Subsequently, cases were stratified to either of the following 3 arbitrary categories according to the underlying spinal pathology, anatomic variations and degree of disturbed alignment. Categorization was performed in agreement by 2 authors specialized in spine surgery.

Category I (normal vertebra anatomy and alignment): vertebral body fractures with intact adjacent vertebras for screw instrumentation (index level spared), minor degenerative disease (Schizas grade A [12]) or minor previous surgery (single level unilateral decompression), normal spinal alignment and no relevant scoliosis (L3 obliquity <15 degrees according to Schwab et al. [13])

Category II (altered vertebra anatomy or alignment): moderate to severe degenerative disease (Schizas grade B-C), extended previous surgeries (multilevel decompression, laminectomy), moderate disturbance of spinal alignment (grade I listhesis according to Meyerding [14]),and moderate lumbar scoliosis (L3 obliquity 15–25 degrees).

Category III (severely altered vertebra anatomy or alignment): severe degenerative disease (Schizas grade D), significant to severe disturbance of spinal alignment (≥ grade II listhesis) and severe lumbar scoliosis (L3 obliquity > 25 degrees).

### 2.2. Image Processing Workflow

For this ex vivo study, initial surgical concepts were extracted from the registry and defined segments addressed and construct length for screw planning in this comparative analysis. All screw planning in this study was performed blinded to previous results on naïve spine CTs (2 mm slice thickness). Screw planning was performed manually by 2 independent raters to create reference screws for each case. Additional screw plans were created by a self-derived planning tool based on deep learning [11] and a commercial atlas-based tool. This analysis was limited to segments L1–L5 for comparison of automatic planning tools in this study.

### 2.3. Manual Planning of Reference Screws

Reference screws were created by2 independent experts in spine surgery with >10 years of surgical experience in navigated instrumentations. For experimental screw planning in this study, we chose the identical setup used during real surgical procedures, which was familiar to both raters (Stryker Spinemap 3D, Stryker, Kalamazoo, MI, USA). Both raters planned screws independently and blinded to previous results. Manual planning created 3D screw segmentation masks representing the desired screw location, trajectory and dimension within the CT data set. Screw parameters were retrieved from the software for further comparison.

### 2.4. Automatic Planning by Self-Derived Approach Based on Deep Learning

We deployed a self-derived tool based on deep learning (DL) for automated screw planning, which was integrated into the open-source software Medical Imaging and Interaction Toolkit (MITK, mitk.org). The tool processes screw planning as an image segmentation task and applies a deep neural network (nnU-Net) on naïve spine CTs as an input volume. Technical details to algorithm development and validation have been reported previously [11,15].

In short, the algorithm was initially trained using 155 spine-CTs with 1052 manually labeled screw trajectories. Using a DL approach, the algorithm learned to derive screw trajectories from the general context of the images provided during training, rather than relying on shape restraints, landmark regression or cortical bone segmentation used in previous approaches [7,8,9,10].

When using the DL algorithm for planning of new cases, desired segments for screw planning are selected by setting vertebra centroids in a graphic user interface and the net proposes 3D segmentation masks representing screw pairs in desired vertebras as a result. Screw parameters (i.e., screw head and tip points, screw direction, length and diameter) used in this study are derived from the paired segmentation masks using connected component filter and principal component analysis for further evaluation described below.

### 2.5. Automatic Planning by a Commercial Atlas-Based Approach

We used a commercial atlas-based (ATL) approach for screw planning available on Brainlab’s Elements Spine & Trauma 3D screw planning app (v1.0.0.172) (Brainlab, Feldkirchen, Germany). On a graphic user interface, the app processed naïve spine CT data and enabled the manual selection of desired vertebras for screw planning, when atlas registration was successful for respective segments. Suggested screws were illustrated as masks in desired vertebras and respective screw parameters were transcribed to the DICOM header information by the app. From the DICOM header, screw parameters were retrieved for further analysis.

### 2.6. Three-Dimensional Quantitative Evaluation of Screw Plans

For evaluation of different screw planning methods, screw plans from DL and ATL were compared to corresponding manually planned screws serving as the reference in this study. 

Since a ground truth definition of an ideal screw position does not exist in the literature, deviations observed between automatically and manually planned screws were evaluated in comparison to the interrater variance of manually planned screws rather than exclusively assessing absolute differences to the manual reference. This sought to test the clinical value of automatic planning tools to replace manual planning. Interrater evaluation was performed for manual screw plans created by rater A vs. rater B. For comparison of screw plans, minimal absolute distances (MAD) were computed for corresponding screw head and tip points (in millimeters) as well as the angular deviation of screw direction (in degrees) in 3D space. MAD was calculated by customizing Python scripts from the NumPy Package (v1.20) as the Euclidean distance between 2 points in 3D space (*x*, *y*, *z* coordinates and their differences *d*) according to the following formula (Formula (1)):

(1) $$MAD=\sqrt{{d(x)}^{2}+{d(y)}^{2}+{d(z)}^{2}}$$

### 2.7. Qualitative Evaluation of Screw Plans

In a screw-by-screw analysis, all results from automatic and manual screw planning were evaluated according to the Gertzbein–Robbins Classification (GR) in agreement by 2 authors specialized in spine surgery. The Gertzbein–Robbins Classification grades the positioning of pedicle screws in relation to cortical bone margins as within (grade A) and <2 mm (grade B), <4 (grade C), <6 mm (grade D), and ≥6 mm (grade E) cortical breach, respectively [16]. Moreover, the direction of pedicle perforations (medial, lateral, superior, inferior) was recorded as previously described in all non-GR grade A screws [17,18]. Anterior screw breaches and screw violation of proximal facet joints were rated separately on a binary scale [19]. All screws scored GR grade A or B were rated clinically acceptable for implantation, whereas screws scored GR grade C, D or E as well as all anterior screw breaches and proximal facet violations were deemed to require a revised plan prior to implantation.

### 2.8. Statistics

All continuous variables from quantitative screw evaluation were evaluated by their means and standard deviation. Normality distribution was tested by the Shapiro–Wilk test and nonparametric comparisons were chosen in absence of normally distributed data. Kruskal–Wallis tests followed by Dunn’s post-test for multiple comparisons was used for evaluation of intergroup variances in quantitative analysis and evaluation of MADs in different spine pathology categories. Selected intergroup differences of quantitative screw measures and qualitative GR grades were evaluated by the t-test. Distributions of qualitative measures across different planning tools were assessed with the X^2^ or Fisher’s exact test. *p*-values <0.05 were regarded as statistically significant. Data composition was performed using Excel (Microsoft Corp., Redmond, WA, USA) and the statistical analysis was performed with Graph Pad Prism 9 (GraphPad Software, San Diego, CA, USA).

## 3. Results

In total, 256 screws in 50 randomly selected cases were evaluated in this study covering levels L1–L5. Median construct length covered 3 vertebras (i.e., 6 screws) (range 2–5 vertebras). Stratification of cases according to spinal pathology resulted in 18 cases (36%) with normal vertebra anatomy and spinal alignment (category I), 16 cases (32%) with altered (category II) and 16 (32%) with severely altered vertebra anatomy or spinal alignment (category III) composing the cohort, respectively. Table 1 overviews descriptive data. DL planning was successful in all targeted 256 screws (100%) in this study, while with ATL, planning was successful in 208 screws (81%). ATL planning failed to produce screw suggestions in 48 screws (19%), which affected planning in 12 cases. Since a quantitative evaluation of automatic screws towards manual reference screws could only be performed when automatic planning produced screw results, this led to an imbalance of DL and ATL subgroups in the following analysis.

### 3.1. Quantitative Evaluation of Screw Plans

In 3D quantitative evaluation, successfully planned screws by DL (*n* = 256, 100%) and ATL (*n* = 208, 81%) were compared to manually planned reference screws and evaluated according to the interrater variance of manual planning.

**Figure 1:**
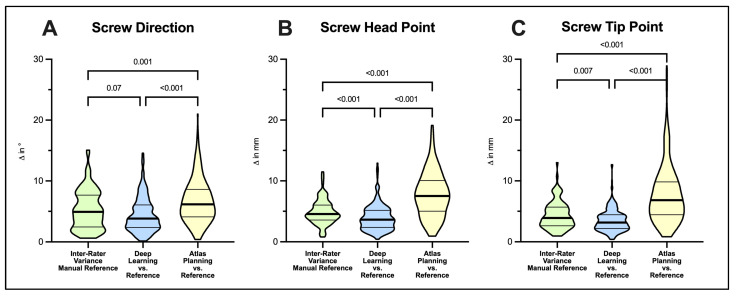
Comparison of screw planning results using screw direction (**A**), screw head (**B**) and screw tip points (**C**) as benchmarks. Violin plots illustrate mean absolute differences for deep-learning- and atlas-based planning tools with their respective manually planned reference screws. Interrater variance of manual screw planning puts automatic planning performance into clinical context of current standards in navigated spine instrumentation. Note the reduced success in atlas-based planning (208/256 screws, 81%) available for analysis. Statistical significance was tested with Kruskal–Wallis tests followed by Dunn’s post-test for multiple comparisons.

The quantitative evaluation is summarized in Table 2 and illustrated in Figure 1.

Evaluating screw trajectories, we observed a mean 5.27 ± 3.20° interrater variance in manual planning, which was statistically comparable to a mean 4.46 ± 2.86° deviation observed in DL when compared to manually planned reference screws (*p* = 0.07). In contrast, ATL-planned screws exhibited a significantly greater 6.70 ± 3.53° mean deviation compared to the interrater variance of manually planned screws (*p* < 0.001) (Figure 1A).

Mean interrater variance for screw head points was 4.89 ± 2.04 mm in manual screw planning. In comparison, mean deviation of DL to reference screw head points was significantly smaller (3.93 ± 2.08, *p* < 0.0001) while ATL-planned screws showed significantly greater deviations from the manual reference (7.77 ± 3.65 mm, *p* < 0.0001), respectively (Figure 1B).

A comparable observation was made for screw tip points. Mean interrater variance was 4.36 ± 2.25 mm in manual planning. DL planning resulted in significantly smaller mean deviations to reference screws (3.49 ± 1.80 mm, *p* = 0.007), while deviations in ATL planning significantly exceeded ranges observed for interrater variance (7.81 ± 4.75 mm, *p* < 0.001) (Figure 1C).

**Figure 2:**
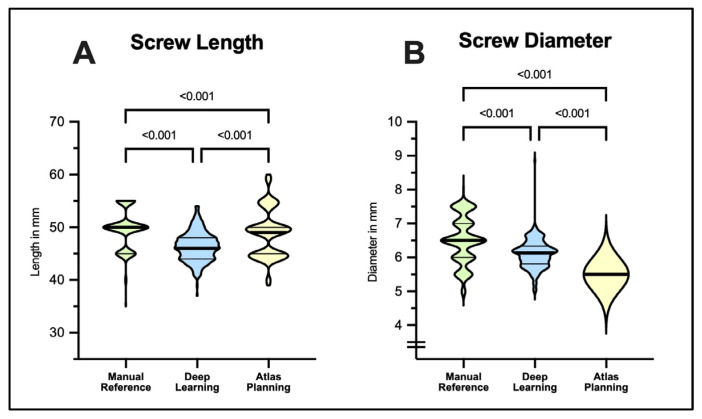
Performance of deep-learning- and atlas-based tools in comparison to manual screw planning by spine surgery experts. Violin plots illustrate proposed values for screw length (**A**) and screw diameter (**B**). In atlas-based planning, screw diameter was set to 5.5 mm by default in all cases analyzed. Note the reduced success in atlas-based planning (208/256 screws, 81%) available for analysis. Statistical significance was tested with Kruskal–Wallis tests followed by Dunn’s post-test for multiple comparisons.

For screw length and diameter, mean absolute values were compared (Figure 2). Manually planned reference screws had a mean length of 49.65 ± 3.80 mm, while both DL and ATL produced significantly shorter screws (46.36 ± 2.70 mm, 48.79 ± 4.51 mm, *p* < 0.001), respectively. Mean screw diameter was 6.51 ± 0.68 mm in manual reference screws while DL suggested screws were significantly thinner (mean 6.10 ± 0.42 mm, *p* < 0.001). ATL screws were set to a standard of 5.50 mm by default without case-specific adjustment, which was also significantly thinner compared to the reference (*p* < 0.001).

### 3.2. Qualitative Evaluation of Screw Plans

Screw fit within the pedicle was evaluated using the Gertzbein–Robbins classification. Manually planned screws all met GR grade A, setting the reference in this study. DL planning produced 249 GR-A screws (97%) and showed minor cortical breach (<2 mm, GR-B) in the remaining 7 screws (3%).

ATL planning proposed 167 GR-A (65%), 29 GR-B (11%), 4 GR-C (2%), 6 GR-D (2%) and 2 GR-E screws (1%) and failed in 48 screws (19%), respectively. This illustrates a considerably higher variance of planning results in ATL compared to DL planning (*p* < 0.0001 vs. non-GR-A screws). Table 3 summarizes qualitative evaluation of screw planning.

Regarding the direction of screw breaches, DL exhibited 2 lateral, 2 medial, 1 inferior and 2 superior breaches, respectively. ATL showed 11 lateral, 4 medial, 22 inferior and 4 superior breaches, respectively. Bilateral breaches affecting both pedicles at a single vertebra were significantly more frequent in ATL (30/41 screws, 73%) compared to DL (2/7 screws, 29%) (*p* = 0.03). The L5 segment was predominantly affected by breaches in 23/41 screws (56%) in ATL and in 7/7 screws (100%) in DL planning. Anterior screw breaches penetrating the anterior vertebral wall were observed frequently in ATL planning in 89 screws (35%) while this was not observed in DL planning. In contrast, proximal facet violations were only observed in DL but not in ATL planning and affected 15 screws (6%). Proximal facet violations were observed predominantly at L5 (12/15 screws, 80%), underscoring L5 as a particular challenge for automatic planning.

Since anterior penetrations carry the risk of large vessel injury and proximal facet violations can contribute to adjacent segment degeneration [20], automatic screw suggestions exhibiting either of these characteristics, or those rated GR-C or worse, were deemed to require a revised plan prior to implantation. Accordingly for DL, 15 screws (6%) required revision due to proximal facet violations. In ATL planning, 141 screws (55%) required revision for GR violations (*n* = 12) and/or anterior breaches (*n* = 89) or needed new manual planning due to failure of automatic planning (*n* = 48). ATL required significantly more revisions of planning compared to DL (*p* < 0.0001).

### 3.3. Robustness of Planning Tools to Anatomic Alteration

**Figure 3:**
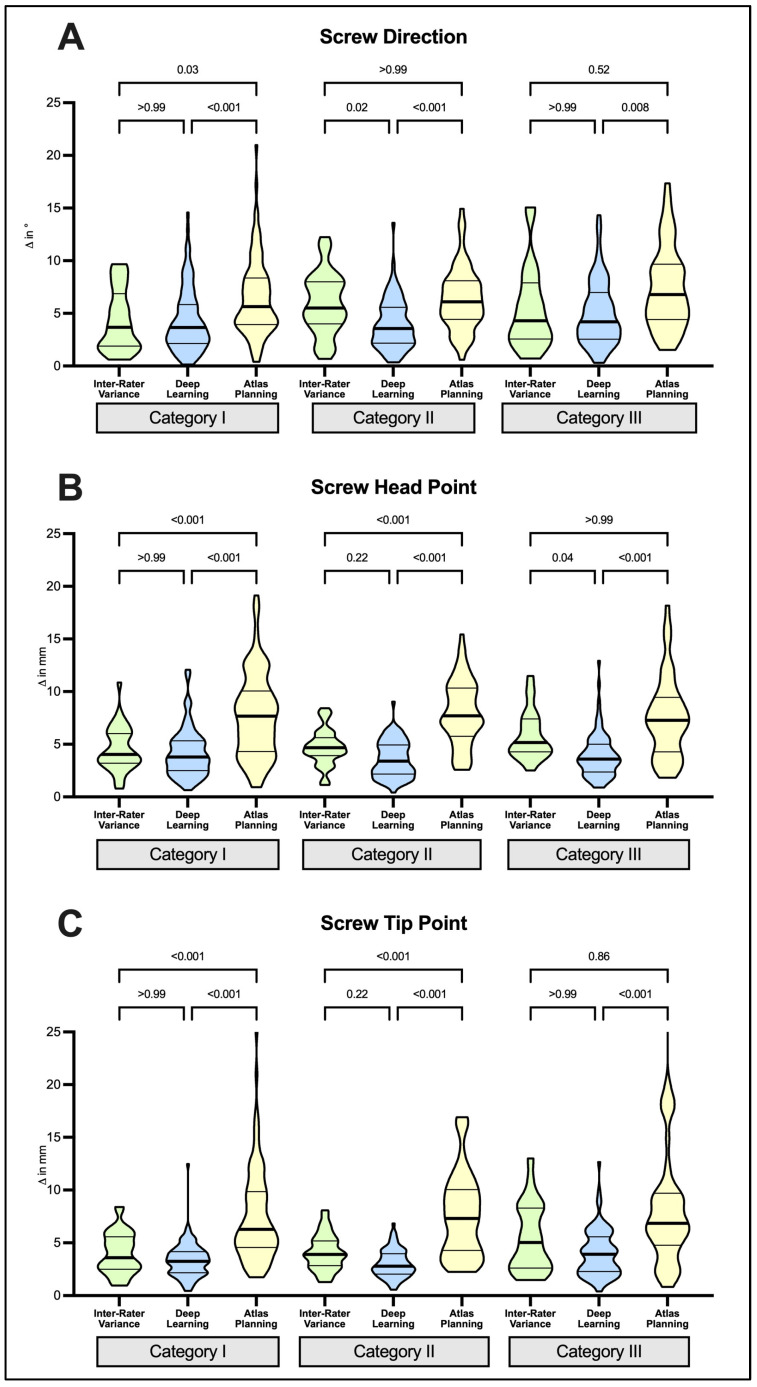
Comparison of screw planning results under consideration of the degree of alteration of vertebra anatomy and spinal alignment. Screw direction (**A**), screw head (**B**) and tip points (**C**) were used as benchmarks for comparison of mean absolute differences between deep-learning- and atlas-based screws with their respective manually planned reference screws and interrater variance of manual screw planning. Categories I–III reflect subgroups of cases with increasing alterations to vertebra anatomy and spinal alignment. Note the reduced success in atlas-based planning (208/256 screws, 81%) available for analysis. Statistical significance was tested with Kruskal–Wallis tests followed by Dunn’s post-test for multiple comparisons.

When analysis was performed in subgroups stratified for the amount of anatomic alteration and disturbed spinal alignment (i.e., categories I–III in Figure 3), different performances were observed for DL- and ATL-based planning tools. 

DL-planned screws remained comparable to manually planned screws in all three subgroups of increasing alteration to spinal anatomy, which was illustrated by mean deviations which either matched or undercut interrater variance of manual planning in screw direction, head and tip point analysis, respectively (Figure 3A–C). This illustrates the particular robustness of DL to increasing anatomic variation and alteration of spine alignment depicted by categories I–III in this study.

In contrast, we observed a marked susceptibility of ATL planning to alterations of spinal anatomy. We observed planning failure in 48 screws (19%) affecting 12 cases. Failed planning occurred in 2/18 category I (17%), 3/16 category II (25%) and was significantly pronounced in in 7/16 category III cases (58%), respectively (*p* = 0.03). Accordingly, planning failed in 10/100 screws (10%) in category I, in 10/74 screws (14%) in category II and was significantly pronounced in 28/82 screws (34%) in category III (*p* < 0.0001). ATL planning failure described above produced bias on quantitative results in subgroup analysis since only successfully planned screws could be compared. Hence, an increase of screw deviation directly correlating with the degree of alterations to spinal anatomy could not be observed (Figure 3A–C).

**Figure 4:**
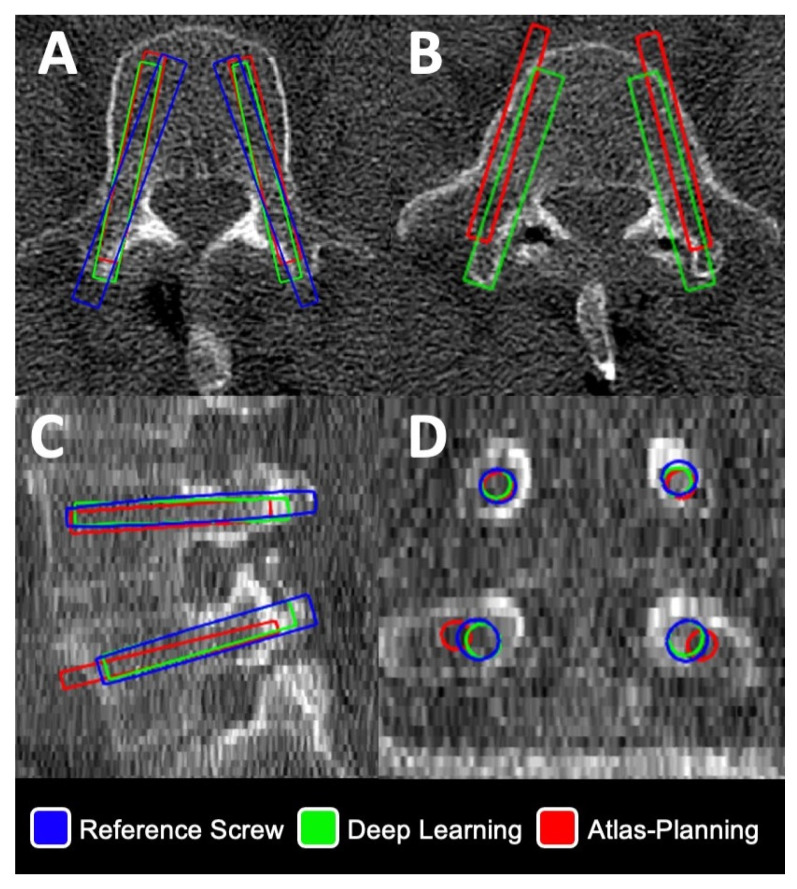
Illustration of virtual screw planning and evaluation for a L4 + L5 instrumentation in axial (**A**,**B**), sagittal (**C**) and coronal (**D**) planes in a category I case with normal vertebra anatomy. Note an anterior screw breach in atlas-based planning (**B**,**C**). Deviations of screw head and tip points and angular deviation were automatically calculated in Python for deep-learning-(green outline) and atlas-based screws (red outline) vs. corresponding reference screws (blue outline), respectively.

**Figure 5:**
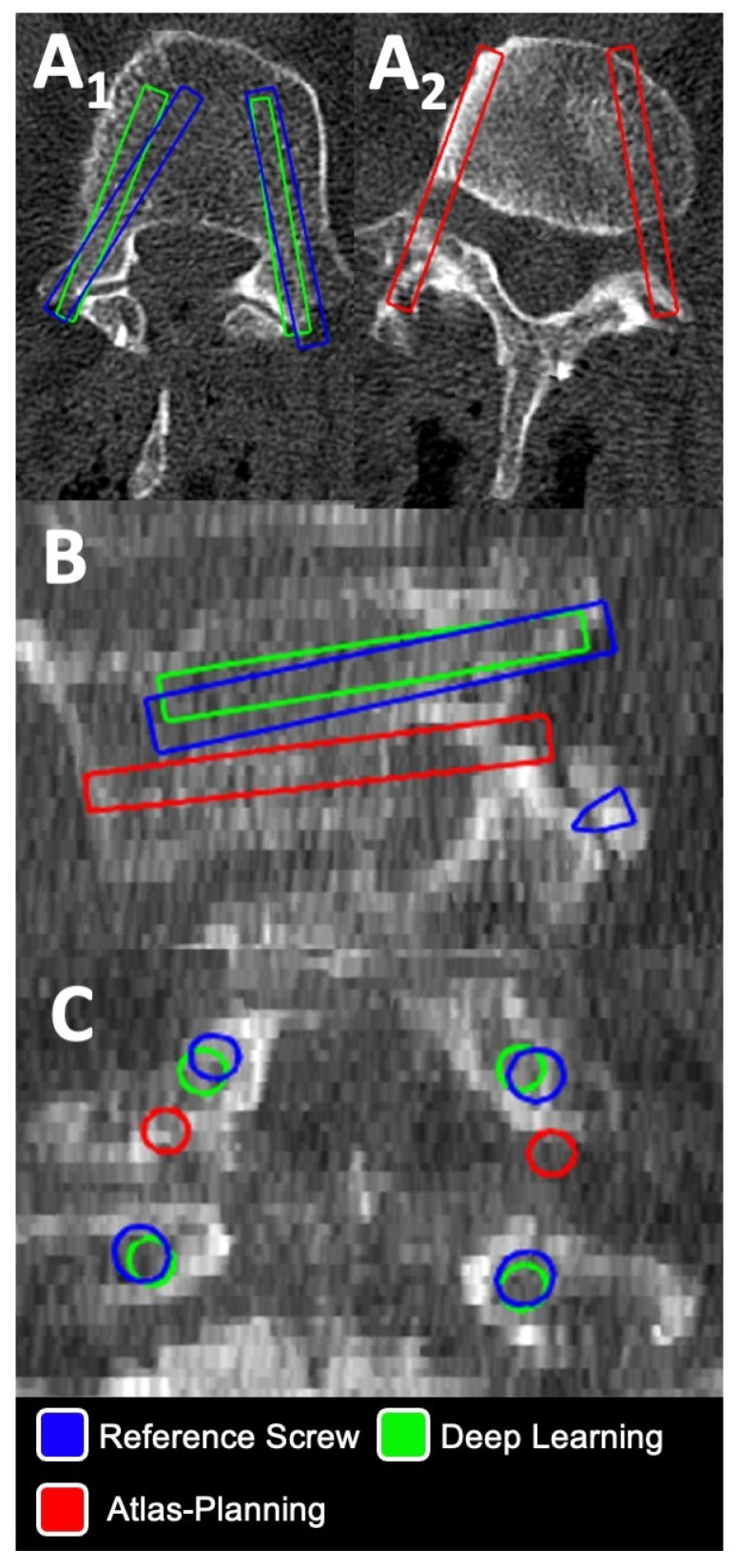
Illustration of screw evaluation in a L4 + L5 instrumentation after previous L5 laminectomy in axial (**A_1_**,**A_2_**), sagittal (**B**) and coronal (**C**) planes. Note the significant pedicle breach in atlas-based planning at L4 (**A_2_**,**B**,**C**). Deviations of screw head and tip points and angular deviation were automatically calculated in Python for deep-learning-(green outline) and atlas-based screws (red outline) vs. corresponding reference screws (blue outline), respectively.

Figure 4 and Figure 5 provide illustrative cases for evaluation of screw planning accuracy evaluated in this study.

Illustrative Case I (Figure 4): Planning for a L4 + L5 instrumentation in axial (A+B), sagittal (C) and coronal (D) planes in a category I case with normal vertebra anatomy. While planning was concordant at L4 (A), an anterior breach occurred in atlas-based planning and facet involvement was observed in deep learning at L5 (B).

Illustrative Case II (Figure 5): In this L4 + L5 instrumentation after a previous L5 laminectomy, acceptable screw suggestions were made by deep learning (Figure 5(A_1_)). Atlas-based planning exhibited a bilateral offset at L4 causing a caudal pedicle breach (Figure 5(A_2_)) suggesting an image-to-atlas registration error. Additionally, atlas-based planning failed at L5 in this case, illustrating susceptibility to anatomic alteration likely attributable to previous laminectomy in this category II case with altered vertebra anatomy.

## 4. Discussion

In this ex vivo study we simulated screw planning workflows for lumbar instrumentations and evaluated a self-derived DL-based approach and a commercial ATL-based approach to automatic screw planning. We found that DL-proposed screws could be regarded noninferior to the manual reference, while screw suggestions made by the ATL tool would require manual adjustments prior to implantation in up to 55% of cases. Alterations to spine anatomy and alignment critically affected ATL results while robust planning was observed in DL. 

In this study evaluating automatic planning under consideration of actual spine pathology, we focused on clinical applicability of the respective tools. To this end, we calculated absolute differences for automatically planned screws and their corresponding reference screws but additionally put observed differences in perspective of the interrater variance of manual planning when performed by two independent spine surgeons. Interrater variance (e.g., mean 5.27 ± 3.20° for screw direction) in our study was comparable to respective means 8.3 ± 7.5° and 3.2 ± 4.3° for sagittal and axial variance, respectively, previously reported in thoracic screws [21]. In absence of a universal gold standard for screw planning, this aimed at evaluating the noninferiority of automatic compared to manual planning [22].

### 4.1. Atlas-Based Planning

Various approaches to automatic pedicle screw planning have been described and atlas-based tools have most frequently been used and evaluated in the literature. In principle, accurate coregistration of case-specific image data to the atlas is crucial in this approach to derive conclusive screw information. While published series on ATL tools frequently rely on normal patient anatomy or consist of small cohorts, anatomical variations and altered alignment of vertebras post typical constraints to registration accuracy, consequently affecting the accuracy of screw suggestions in previous studies [7,8,9]. While machine-learning approaches have been shown to improve image-to-atlas coregistration, spinal deformity, vertebral degeneration and alterations attributable to previous surgeries persistently pose challenges leading to failure of ATL-based planning [10]. 

In our study, the ATL-based tool exhibited limitations attributable to its underlying approach, translating into deviations from reference screws, which exceeded interrater variance for manual planning and led to the necessity for manual revision of screw suggestions in 55% of cases (Table 2 and Table 3). Particularly, anterior screw breaches were an issue in 35% of ATL-planned screws (Figure 4B). Given concordant deviations from the reference found for head and tip points (7.77 ± 3.65 mm and 7.81 ± 4.75 mm, respectively) along with the clinically insignificant difference in suggested screw length of <1 mm, this could reflect issues in atlas coregistration, imposing a positional offset on an otherwise correctly dimensioned screw. In cases with pedicular screw breaches (41/256 screws, 16%), this affected both pedicles of a segment in 73% of respective cases in ATL planning. This points in the same direction, suggesting an image-to-atlas offset triggering bilateral screw misplacement (Figure 4B and Figure 5(A_2_)). Since a commercial system was used for ATL planning in this study, no data on coregistration accuracy achieved in our cohort could be extracted for further analysis or comparison to data from the literature [8,10].

### 4.2. Deep-Learning-Based Planning

Our group previously described a novel deep-learning-based approach to pedicle screw planning, which implicitly learned screw placement from a large reference data set of expert labeled screws [11]. In line with our results from the initial publication, we achieved comparable screw accuracy results in the current cohort for DL planning regarding overall performance in 3D-quantitative evaluation of respective screw directions, head and tip points and also qualitative screw evaluation according to GR. This corroborates our initial finding that DL-based planning can be regarded as noninferior to manual planning in a total of 386 screws in two randomly selected surgical cohorts [11] (Figure 4 and Figure 5). Nevertheless, we determined DL plans to require revision in 15 screws (6%) of cases due to violations of the proximal facet joints, which predominantly affected the L5 vertebra in 12/15 screws (80%) in our current study (Figure 4B). The segmental accentuation of misplacement hints at an inherited error originating from biased initial training data of the algorithm, which is a known issue in training of deep-layered networks [23]. Training data was derived from real surgical planning data, where screw placement in the vicinity of the facet at L5 was individually tolerated reflecting the surgical decision weighting an ideal screw trajectory against technical feasibility and invasiveness during the procedure [11]. While the clinical relevance of isolated facet violations is equivocal, its association to adjacent segment degeneration should lead to a refinement of the DL algorithm to eliminate this sole issue triggering revisions in this series and to carefully spare facets in the future [20].

Even though current DL planning results are promising, the decision process of the algorithm towards screw suggestions cannot be reconstructed. Since this is a common issue for appliance of deep-learning in medicine, future work should focus on explainability measures to develop insights into the decision process and to reassure results for surgical decision making [24].

### 4.3. Greater Robustness of DL Method

The evaluation of susceptibility of automatic planning tools to different amounts of altered spinal anatomy and alignment was a focus of this study. From technical aspects, DL-based approaches are expected to show greater robustness to structural variations of the vertebras, since DL does not rely on explicitly defined constraints, the identification of geometric components or atlas-based modeling [8,9,25,26]. Our findings could corroborate this hypothesis showing robust screw proposals throughout all three categories of altered spinal anatomy encompassing degenerative disease, spine fractures, spondylolisthesis and degenerative deformity of the lumbar spine (Figure 3). 

For ATL planning, we anticipated an increase of screw deviations from the reference along with increasing anatomic complexity. However, we observed screw suggestions of comparable accuracy throughout all subgroups. This can be explained by a positive selection bias after failure of a substantial number of screws, particularly affecting quantitative results in category III with highest anatomic complexity (Figure 3). This led to exclusive analysis of successfully planned screws, which mitigated possible differences between subgroups. Nevertheless, pronounced rates of planning failure in category III unveiled the marked susceptibility of ATL-based planning in our study, which reflected known limitations of the methodology [8,9,21,25,26] (Figure 5).

Failure of automatic screw planning, along with screw breaches, led to a significantly higher necessity to manually revise screws in ATL. After all, this particularly accounted for the poorer performance in comparison to DL in this analysis. 

Mutual restrictions were observed for both methods at L5, causing all observed screw breaches in DL (7/7) and 23/41 (56%) of breaches in ATL. This illustrates the unique challenges of automatic planning for this segment. To improve performance at this level for DL, the training of a segment-specific model could be evaluated instead of the general lumbar spine model currently applied in the algorithm.

### 4.4. Limitations

Our study has limitations originating from its ex vivo design and experimental nature. First, reported results were based on a representative but small data set of lumbar screws, which limits the generalizability of our results to lumbar instrumentations or other spine segments in general. To perform planning as close to reality as possible, we selected cases from a retrospective database and adopted the initial surgical concepts for simulated planning. Both surgeons involved in manual planning were highly familiarized with navigated instrumentation. While this warranted high quality manual planning in our study, interrater variance may be higher than reported in other surgeons and/or setups [21]. 

This study sought to incorporate different levels of anatomical complexity to evaluate robustness of automatic planning tools. While the stratification performed in this analysis was sufficient to generally elucidate increased robustness of DL over ATL towards anatomical variation, heterogeneity of pathologies and variation of surgical concepts with regards to the number of spinal segments addressed were a limitation to generalizability of our results. Specifically, the impact of certain conditions (e.g., increasing degree of spondylolisthesis) on screw planning accuracy could not be further quantified for both automatic planning tools in this study. Further evaluation in matched cohorts for different degrees of specific spine diseases is needed to corroborate data on robustness found in this study.

## 5. Conclusions and Outlook

ATL-based planning was able to produce screw suggestions that frequently needed manual corrections prior to implantation due to methodological constraints, making ATL-based approaches susceptible to anatomic variability. Nevertheless, ATL can contribute to more efficient surgical workflows by expediting the planning process in comparison to manual screw planning from scratch.

DL-proposed screws were comparable to expert-planned screws and exhibited higher general robustness to anatomic alteration in this study. This makes DL a highly promising approach to fully automate planning for navigated or robotic spine procedures, where the remaining manual interaction could be the confirmation of generated screw plans by the surgeon for liability reasons.

Beyond that, the DL framework enables incorporation of additional neural layers to enhance various aspects of screw planning. For instance, consideration of local bone mineral density could be used for optimization of screw dimensions and trajectory to maximize fastening strength. This could further translate into improved clinical results based on optimized and patient-specific surgical planning in the future [7,27].

## Figures and Tables

**Table 1:** Descriptive Data.

Variable	*n*	%
**Selected Cases**	**50**	**100**
**Spine Pathology Category**		
**Category I** (normal vertebra anatomy and spinal alignment)	18	36
**Category II** (altered vertebra anatomy or spinal alignment)	16	32
**Category III** (severely altered vertebra anatomy or spinal alignment)	16	32
**Construct Length (Vertebras)**		
2	20	40
3	16	32
4	13	26
5	1	2
**Planned Screws**	**256**	**100**
L1	2	1
L2	18	8
L3	54	21
L4	88	34
L5	94	36

**Table 2:** Quantitative Screw Evaluation.

	Interrater Variance Manual Planning	Deep-Learning Planning	(vs. Manual Planning)	Atlas-Based Planning	(vs. Manual Planning)
Mean ± S.D.			***p*-Value**		***p*-Value**
**Screw Direction** (Δ in degree)	5.27 ± 3.20	4.46 ± 2.86	0.07	6.70 ± 3.53	**0.001**
**Screw Head Point** (Δ in mm)	4.89 ± 2.04	3.93 ± 2.08	**<0.001**	7.77 ± 3.65	**<0.001**
**Screw Tip Point** (Δ in mm)	4.36 ± 2.25	3.49 ± 1.80	**0.007**	7.81 ± 4.75	**<0.001**
**Screw Length** (absolute in mm)	49.65 ± 3.80	46.36 ± 2.79	**<0.001**	48.79 ± 4.51	**<0.001**
**Screw Diameter** (absolute in mm)	6.51 ± 0.68	6.10 ± 0.42	**<0.001**	5.50 ± 0.00	**<0.001**

Δ calculated as deviation to manual reference screws for DL and ATL, respectively and as interrater variance between independent raters for manual planning. Significance (*p* < 0.05) is illustrated in bold face.

**Table 3:** Qualitative Screw Evaluation.

*n* = 50 Cases *n* = 256 Screws	GR-Grade A	GR-Grade B	GR-Grade C	GR-Grade D	GR-Grade E	Failed Planning	Anterior Breach	Proximal Facet Violation	Screws Requiring Revision
**Manual** **Planning**	512 * (100%)	0	0	0	0	0	0	0	0
**Deep-Learning** **Planning**	249 (97%)	7 (3%)	0	0	0	0	0	15 (6%)	15 (6%)
**Atlas-Based** **Planning**	167 (65%)	29 (11%)	4 (2%)	6 (2%)	2 (1%)	48 (19%)	89 (35%)	0	141 (55%)

GR: Gertzbein–Robbins Grade, * Reference screws were planned independently by two experts in spine surgery.

## Data Availability

The data that support the findings of this study are available on request from the corresponding author. The data are not publicly available due to contained information compromising privacy necessitating informed consent. Intellectual property of the DL screw planning algorithm used in this study is held by the authors Lisa Kausch, Klaus Maier-Hein and Moritz Scherer.
